# DeepLontar dataset for handwritten Balinese character detection and syllable recognition on Lontar manuscript

**DOI:** 10.1038/s41597-022-01867-5

**Published:** 2022-12-10

**Authors:** Daniel Siahaan, Ni Putu Sutramiani, Nanik Suciati, I Nengah Duija, I Wayan Agus Surya Darma

**Affiliations:** 1 Department of Informatics, Faculty of Intelligent Electrical and Informatics Technology, Institut Teknologi Sepuluh Nopember, Surabaya, 60111 Indonesia; 2 Department of Information Technology, Faculty of Engineering, Universitas Udayana, Badung, 80361 Indonesia; 3 Department of Balinese Language Education, Postgraduate, Universitas Hindu Negeri I Gusti Bagus Sugriwa, Denpasar, 80236 Indonesia; 4 Department of Informatics, Faculty of Technology and Informatics, Institut Bisnis dan Teknologi Indonesia, Denpasar, 80225 Indonesia

**Keywords:** Scientific data, Science in culture, Computer science

## Abstract

The digitalization of traditional Palmyra manuscripts, such as Lontar, is the government’s main focus in efforts to preserve Balinese culture. Digitization is done by acquiring Lontar manuscripts through photos or scans. To understand *Lontar*’s contents, experts usually carry out transliteration. Automatic transliteration using computer vision is generally carried out in several stages: character detection, character recognition, syllable recognition, and word recognition. Many methods can be used for detection and recognition, but they need data to train and evaluate the resulting model. In compiling the dataset, the data needs to be processed and labelled. This paper presented data collection and building datasets for detection and recognition tasks. *Lontar* was collected from libraries at universities in Bali. Data generation was carried out to produce 400 augmented images from 200 Lontar original images to increase the variousness of data. Annotations were performed to label each character producing over 100,000 characters in 55 character classes. This dataset can be used to train and evaluate performance in character detection and syllable recognition of new manuscripts.

## Abstract (summary)

**Table:** 

Measurement(s)	accuracy • Precision • Recall • F1-score
Technology Type(s)	Python
Factor Type(s)	bounding box • frequency • spatial location • dimensional size
Sample Characteristic - Organism	lontar • manuscript • glyphs
Sample Characteristic - Environment	balinese glyphs
Sample Characteristic - Location	Bali Province

## Background & Summary

Ancient manuscript digitization is a necessary process to support the preservation of cultural heritage to avoid document destruction. The digitization process is carried out through the acquisition of ancient manuscript documents into digital images. Then, digital images can be further processed through the computer vision method to extract the information in the ancient manuscript document. Balinese *Lontar* manuscript is a historical document used by ancient people in Bali to store important information related to ancient science, such as traditional medicine, farming techniques, determining auspicious days, and others.

In the ancient Balinese community, traditions, instructions, and drugs ingredients were documented by officials or scholars as *Lontar* manuscripts in Balinese characters. The writing process on the Balinese *Lontar* manuscript uses a special knife called a *pengrupak* on dried palm leaves. Then, roasted candlenut powder is used to give colour to the written Balinese characters. Balinese writers did the writing of *Lontar* to store various important information in ancient times. The Balinese characters used have unique writing characteristics. Characters are written without spaces. There are combinations of characters to form syllables, dense and overlapping characters, and sticking together. DeepLontar dataset can be used for syllables recognition by combining each character by applying special rules. This dataset is very challenging because it can only be read and translated by experts.

Balinese *Lontar* publicly available datasets are available on a very limited basis. Therefore, related research has been carried out for assembling datasets for Balinese *Lontar* manuscripts. Windu *et al*.^1^ proposed AMADI_LontarSet that consists of bi-level images as gold standard dataset, image datasets with word-level annotations and isolated glyphs. The resulting performance is only below 50% due to the use of isolated character images, which do not label every character in the Balinese *Lontar* manuscript. Other studies related to Balinese characters have been carried out, starting with Balinese character segmentation^2^, Balinese character recognition^3^, Balinese character augmentation in increasing data variation^4^, and Balinese character detection based on deep learning^5^. In the case of ancient Chinese documents, two main datasets were proposed. The datasets were annotated with characters, including gold-standard character bounding boxes and its corresponding glyphs^6^. Furthermore, a new augmentation method was introduced based on the fusion of general transfiguration with local deformation and successfully enlarged the training dataset^7^. In the case of Indian documents, thorough experimentations were performed on other corpus comprising in print and in-writing texts^8^. Other studies proposed the IFN/ENIT dataset to surmount the dearth of Arabic datasets easily accessible for researchers^9^ and a popular literature Arabic/English dataset: Everyday Arabic-English Scene Text dataset (EvArEST) for Arabic text recognition^10^. Other researchers proposed Ekush dataset for Bangla handwritten text recognition^11^, Tamil dataset for in-writing Tamil character recognition utilizing deep learning^12,13^, DIDA dataset for detection and recognize in-writing numbers in ancient manuscript drawings dated from the nineteen century^14^.

Based on previous research, we proposed DeepLontar, a dataset for handwritten Balinese character detection and syllable recognition on the Lontar manuscript. DeepLontar consists of 600 images of the Balinese *Lontar* manuscript that have been annotated and validated by experts. This dataset was built through the process of acquisition (200 original images), data generation (400 augmented images), data annotation, and expert validation. This dataset has been tested on the detection and recognition process of Balinese characters using the YOLOv4 model. The original dataset was split into train and test data with distribution ratio of 60%:40%. Three datasets were prepared. The first dataset, i.e. the original dataset, was split into 120 original images in the train data and 80 original images in the test data. In the second dataset, 200 augmented images (produced by the grayscale augmentation technique) were added into the train data. In the third dataset, another 200 augmented images (produced by adaptive gaussian thresholding technique) were added into the train data. In those three dataset, the YOLOv4 model produces a detection performance with mean average precision (mAP) of up to 99.55% with precision, recall, and F1-score are 99%, 100%, and 99%, respectively^5^. DeepLontar consists of 55 Balinese character classes. These classes are used in writing Balinese script in Lontar Manuscripts. The entire vocabulary in the DeepLontar dataset uses these 55-character classes. DeepLontar have been annotated and validated by experts.

**Fig. 1:**
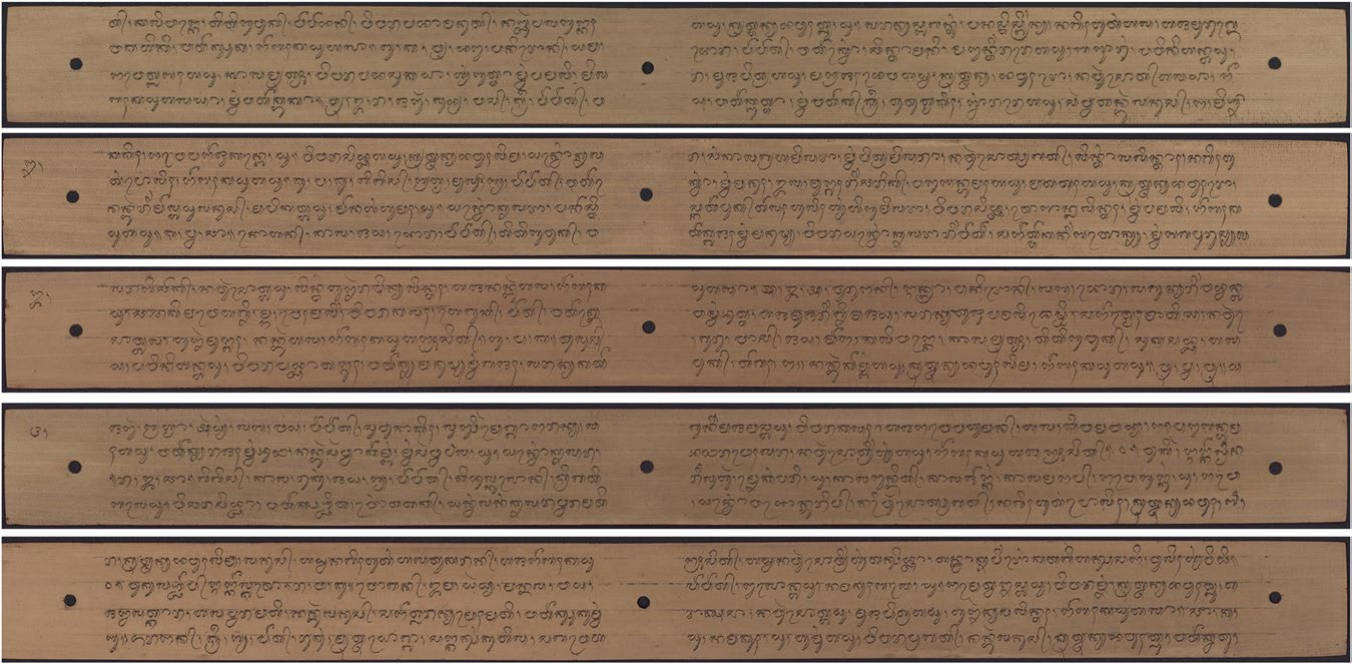
Sample of lontar manuscript.

Each annotated character class has a high variation because it is written using a *pengrupak*, and the characters are handwritten. The high character variation makes this dataset very challenging for detecting and recognizing syllables in Balinese *Lontar* manuscripts. Figure 1 shows a sample image of Balinese lontar manuscript. The Balinese character classes that have been annotated in the Balinese *Lontar* manuscript.

The lontar manuscripts are written using Balinese characters. The writing uses a special knife called *pengrupak* by scraping dry palm leaves so that Balinese characters are engraved on the manuscript. The coloring process uses roasted candlenut powder, making the engraved characters black.

**Fig. 2:**
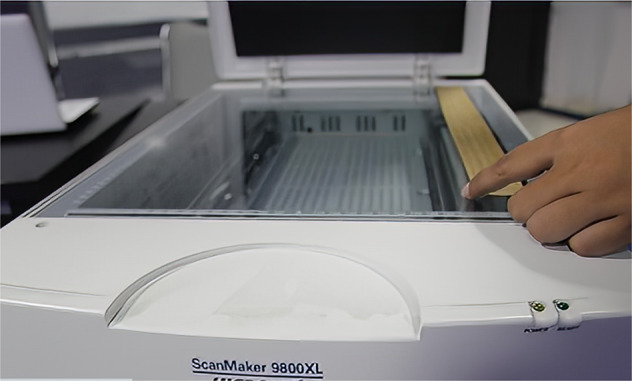
Acquisition of Balinese *Lontar* manuscripts using a scanner.

**Fig. 3:**
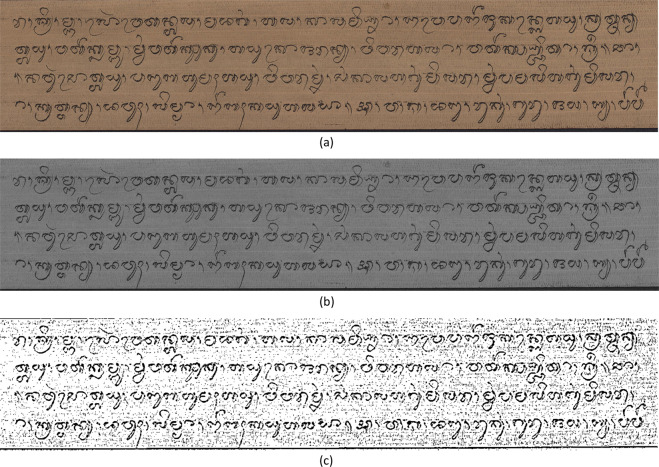
Variations of Balinese *Lontar* manuscript images through the data generation process using data augmentation techniques.

Figure 2 shows the acquisition process of Balinese *Lontar* manuscripts. It is carried out using a scanner. This process is carried out on 200 pieces of Balinese *Lontar* manuscript. To enrich the variety and increase the amount of data, we apply data generation using augmentation techniques. Based on the data generation process, we produced 400 augmented images of the Balinese *Lontar* manuscript. Figure 3 shows variations of the Balinese *Lontar* manuscript image in the DeepLontar dataset.

**Fig. 4:**
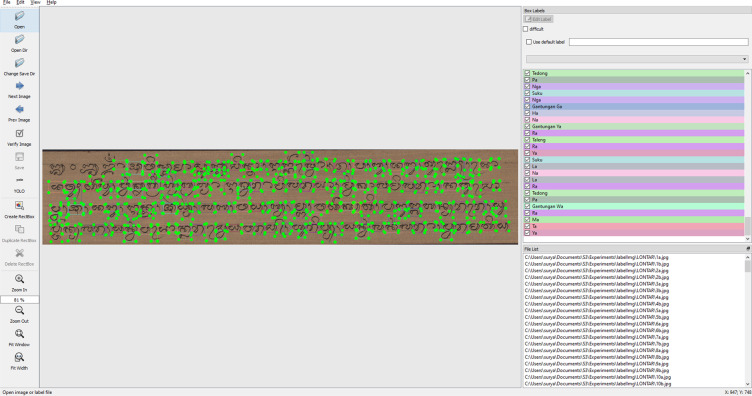
Balinese character annotation on Balinese lontar manuscript using LabelImg and validated by experts.

**Fig. 5:**
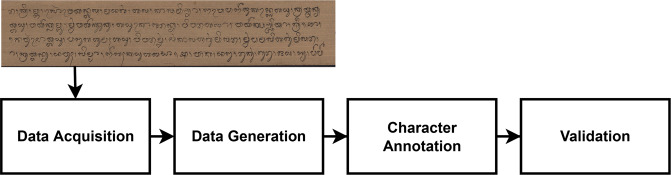
Overview of the processing steps to generate DeepLontar dataset.

**Fig. 6:**
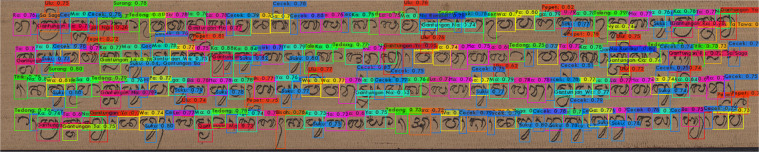
Examples of Balinese character detection and recognition results in DeepLontar dataset.

Figure 4 shows an annotated image of the Balinese *Lontar* manuscript. The annotation process uses LabelImg by labeling each Balinese character. Then, it aims to label the Balinese character class and position in the Balinese *Lontar* manuscript. We have tested the DeepLontar dataset using a deep learning architecture for detecting and recognizing Balinese characters in the Balinese *Lontar* manuscript shown in Fig. 5. In general, each character has been successfully detected, and its class recognized accurately with a confidence level of 99%. Figure 6 Examples of Balinese character detection and recognition results in DeepLontar dataset.

## Methods

The process of compiling the dataset was carried out in four stages. Each stage was shown in Fig. 5, starting with data acquisition, data generation, data annotation, and validation. The first stage was data acquisition by scanning the *Lontar* manuscript using a scanner. Figure 3 shows the scan process per sheet of *Lontar* manuscripts. The *Lontar* manuscript was scanned in a horizontal position according to the characteristics of the elongated *Lontar*. This process produced 200 *Lontar* images. Furthermore, the second stage was to perform data generation with two augmentation techniques. The augmentation technique used grayscale and adaptive gaussian thresholding for increasing the variety of data. The grayscale augmentation is used in order for the model to put lesser importance on colour as a signal. The adaptive gaussian thresholding is utilized to sharpen the character image. This process produced 400 augmented images, which have been enhanced. Overall, the number of initial images and the augmented images of Lontar manuscript was 600 images. Table 1 shows he complete character set of Balinese character classes in DeepLontar dataset. It also shows the average precisions of character detection model trained on original dataset (ori) and trained on augmented dataset (aug). It indicates that the augmentation technique does improve the average precision (AP).

**Table 1:** DeepLontar consists of 55 Balinese character classes and the number of each character classes in DeepLontar.

Character Classes	Freq	AP (%)		Character Classes	Freq	AP (%)	
		Ori	Aug			Ori	Aug
Ha	2,544	99.54	99.88	Gantungan Ga	294	99.33	100.00
Na	5,649	99.44	99.61	Gantungan Ba	255	96.66	97.98
Ca	654	99.53	100.00	Gantungan Nga	177	100	98.51
Ra	3,324	99.76	100.00	Gantungan Pa	384	98.80	100.00
Ka	3,801	99.40	99.88	Gantungan Ja	27	88.89	100.00
Da	1,725	99.72	99.58	Gantungan Ya	1,809	98.70	99.76
Ta	4,401	99.71	99.91	Gantungan Nya	3	100	100.00
Sa	2,625	99.40	99.30	Tedong	2,973	99.47	100.00
Wa	4,128	99.62	100.00	Ulu	5,166	98.85	99.35
La	3,975	99.49	99.85	Suku	8,646	94.64	99.17
Ma	3,408	99.76	100.00	Taleng	2,709	99.95	100.00
Ga	1,350	99.61	99.78	Pepet	2,580	98.11	98.96
Ba	1,896	99.42	99.50	Cecek	13,512	96.57	99.38
Nga	2,538	99.75	100.00	Surang	1,335	98.30	100.00
Pa	2,397	99.68	99.95	Bisah	2,550	99.40	100.00
Ja	849	99.87	100.00	Adeg-adeg	2,403	98.95	99.94
Ya	2,784	99.47	99.70	Titik	1,257	99.60	100.00
Nya	261	96.21	96.63	A Kara	204	100	100.00
Gantungan Ha	615	98.37	99.20	I Kara	141	98.32	100.00
Gantungan Na	417	99.87	99.75	U Kara	231	100	100.00
Gantungan Ca	42	100	100.00	Sa Saga	825	98.72	99.99
Gantungan Ra	1,287	98.84	99.98	Na Rambat	168	99.74	99.98
Gantungan Da	642	91.81	95.49	Da Madu	15	80.00	100.00
Gantungan Ta	450	95.56	97.07	La Lenga	192	99.95	100.00
Gantungan Sa	222	98.17	100.00	Gantungan Da Madu	135	99.23	100.00
Gantungan Wa	1,224	99.54	99.98	Gantungan Ra Repa	636	97.86	99.06
Gantungan La	381	99.40	98.10	Gantungan Ta Tawa	435	99.83	100.00
Gantungan Ma	315	98.16	100.00				

Although DeepLontar dataset does contain out of vocabulary classes, it suffers from imbalance problem. The *da madu* class rarely appear in the dataset. As we can see in Table 1, the augmentation technique helps improves the average precision of the detection model.

The third stage was character annotation using the LabelImg application. The Balinese character originally consists of 75 character classes, but not all character classes are used in writing lontar manuscript. Therefore, to determine the number of character classes, we have involved experts in determining the character classes that are often used in writing *Lontar* manuscript. Image annotation was done to label the image, which was used as ground truth. The bounding box was used to annotate each character. This process was carried out by a team and accompanied by experts. Character annotations produced 102,966 characters came from 55 character classes. The annotation results stored the spatial location of each character object within the observed image. The character class is annotated with the bounding box, its spatial location, and its two-dimensional size. Balinese character annotation in the *Lontar* manuscript produced a new Balinese character dataset for identifying Balinese glyphs called DeepLontar. The last stage was data validation. Based on the result of our experimentation, the dataset was able to produce up to 99.55% performance.

## Data Records

DeepLontar dataset is freely accessible to the researchers at Figshare^15^. DeepLontar consisted of 600 images of Balinese Lontar manuscripts and additionally, 600 *.txt files that stored information related to data annotations in YOLO format. Balinese character annotations in DeepLontar consisted of more than 100,000 characters that experts had validated. All files are named in the following format:

- JPEG images: < filename > .jpg, for instance: 1a.jpg, and
- TXT annotations: < filename > .txt, for instance: 1a.txt,
  Annotation files format follows the YOLO format, as follow:
- <ID> <x> <y> <width> <height>, for instance: 54 0.068000 0.083333 0.016000 0.073333

where <ID> is the object class ID, <x> is x coordinate, <y> is y coordinate, <width> is width of the bounding box, and <height> is heigh of the bounding box. Table 1 shows 55 Balinese character classes.

## Technical Validation

Data validation was carried out in two ways: validation from experts and testing using one of the deep learning methods, namely YOLO. Validation by experts was carried out when making ground truth of Balinese characters in *Lontar* manuscripts. The second validation was a trial with detecting and recognizing Balinese characters using YOLO.

## Usage Notes

DeepLontar dataset images are published and bundled into one compressed file (.zip) named DeepLontar.zip. The annotation files are published and bundled into one compressed file (.zip) named DeepLontar_labels.zip.

## Data Availability

The images data are available at Figshare repository^15^ and data augmentation code are available using OpenCV library. Data annotation tool using LabelImg is available online^16^.
